# Ischaemic postconditioning reduces apoptosis in experimental jejunal ischaemia in horses

**DOI:** 10.1186/s12917-021-02877-y

**Published:** 2021-04-26

**Authors:** Nicole Verhaar, Nicole de Buhr, Maren von Köckritz-Blickwede, Marion Hewicker-Trautwein, Christiane Pfarrer, Gemma Mazzuoli-Weber, Henri Schulte, Sabine Kästner

**Affiliations:** 1 Clinic for Horses, University of Veterinary Medicine Hannover, Hannover, Germany; 2 Department of Biochemistry, University of Veterinary Medicine Hannover, Hannover, Germany; 3 Research Center for Emerging Infections and Zoonoses, University of Veterinary Medicine Hannover, Hannover, Germany; 4 Institute of Pathology, University of Veterinary Medicine Hannover, Hannover, Germany; 5 Institute for Anatomy, University of Veterinary Medicine Hannover, Hannover, Germany; 6 Institute for Physiology and Cell Biology, University of Veterinary Medicine Hannover, Hannover, Germany; 7 Institute of Functional and Applied Anatomy, Hannover Medical School, Hannover, Germany; 8 Small Animal Clinic, University of Veterinary Medicine Hannover, Hannover, Germany

## Abstract

**Background:**

Ischaemic postconditioning (IPoC) refers to brief periods of reocclusion of blood supply following an ischaemic event. This has been shown to ameliorate ischaemia reperfusion injury in different tissues, and it may represent a feasible therapeutic strategy for ischaemia reperfusion injury following strangulating small intestinal lesions in horses. The objective of this study was to assess the degree cell death, inflammation, oxidative stress, and heat shock response in an equine experimental jejunal ischaemia model with and without IPoC.

**Methods:**

In this randomized, controlled, experimental in vivo study, 14 horses were evenly assigned to a control group and a group subjected to IPoC. Under general anaesthesia, segmental ischaemia with arterial and venous occlusion was induced in 1.5 m jejunum. Following ischaemia, the mesenteric vessels were repeatedly re-occluded in group IPoC only. Full thickness intestinal samples and blood samples were taken at the end of the pre-ischaemia period, after ischaemia, and after 120 min of reperfusion. Immunohistochemical staining or enzymatic assays were performed to determine the selected variables.

**Results:**

The mucosal cleaved-caspase-3 and TUNEL cell counts were significantly increased after reperfusion in the control group only. The cleaved-caspase-3 cell count was significantly lower in group IPoC after reperfusion compared to the control group. After reperfusion, the tissue myeloperoxidase activity and the calprotectin positive cell counts in the mucosa were increased in both groups, and only group IPoC showed a significant increase in the serosa. Tissue malondialdehyde and superoxide dismutase as well as blood lactate levels showed significant progression during ischaemia or reperfusion. The nuclear immunoreactivity of Heat shock protein-70 increased significantly during reperfusion. None of these variables differed between the groups. The neuronal cell counts in the myenteric plexus ganglia were not affected by the ischaemia model.

**Conclusions:**

A reduced apoptotic cell count was found in the group subjected to IPoC. None of the other tested variables were significantly affected by IPoC. Therefore, the clinical relevance and possible protective mechanism of IPoC in equine intestinal ischaemia remains unclear. Further research on the mechanism of action and its effect in clinical cases of strangulating colic is needed.

## Background

Strangulating small intestinal lesions in horses can be effectively treated by small intestinal resection. Nevertheless, there is still a need for additional therapeutic strategies, as some cases are not amenable to intestinal resection, and concurrent disease such as post-operative ileus and adhesions are associated with high mortality rates [1, 2]. These complications can result from intestinal damage during ischaemia or due to reperfusion injury [3, 4]. During reperfusion, the formations of reactive oxygen species can induce oxidative injury and neutrophilic inflammation, leading to mucosal and seromuscular damage [2, 5–7]. This may also affect the intestinal neurons, contributing to postoperative motility disorders [8, 9].

Ischaemic postconditioning (IPoC) refers to brief periods of reocclusion of blood supply following an ischaemic event, thereby preventing immediate reperfusion [10]. This principle was first described in the myocardium [11], followed by its application in many tissues including the small intestine, kidney, liver and brain [10, 12]. Most research assessing the effect of IPoC on intestinal ischaemia/reperfusion (I/R) injury, has been performed in laboratory animals by use of an experimental model occluding the cranial mesenteric artery. Many different variables for intestinal ischaemia reperfusion injury have been investigated in these studies, where intestinal IPoC was most commonly associated with decreased histomorphological damage [13–21]. Moreover, reduced apoptosis, less oxidative stress and neutrophil activation, as well as higher superoxide dismutase or glutathion levels have been reported in groups subjected to IPoC [14–21]. It has been suggested that the reocclusion delays the washout of mediators such as adenosine and bradykinin, which may elicit a protective response [10, 22]. Many signalling pathways have been indicated to mediate the effect of IPoC; however, the exact mechanism of action of IPoC remains unknown [12, 22]. Heat shock proteins (HSPs) are molecular chaperones for protein repair that are upregulated in response to a variety of noxious stimuli [23]. Especially the HSP-70 family has been shown to play a protective role in the intestine, with upregulation after intestinal I/R in different animal models [24, 25], and reduced intestinal necrosis associated with higher HSP-70 levels [26]. Furthermore, the upregulation of HSP-70 has been reported after remote conditioning and ischaemic preconditioning in the brain, spinal cord and heart [27–29]. Until now, the role of HSP-70 has not been assessed in tissues undergoing IPoC or in experimental equine jejunal ischaemia.

A recent study investigating IPoC in experimental jejunal ischaemia in horses, found lower mucosal permeability and less epithelial denudation in the group undergoing IPoC [30]. IPoC could represent a feasible technique for clinical cases of strangulating colic; however, more information on the effect of IPoC in the ischaemic equine intestine is needed. The objective of this study was to assess the degree of cell death, inflammation, oxidative stress, and heat shock response during experimental small intestinal ischaemia in horses subjected to IPoC and horses undergoing normal reperfusion. We hypothesized that IPoC would decrease apoptosis, inflammation, neuronal cell death, and oxidative stress in the equine intestine after I/R injury. Furthermore, we hypothesised that IPoC would be accompanied by an increased heat shock response.

## Results

### Cell apoptosis

**Fig. 1:**
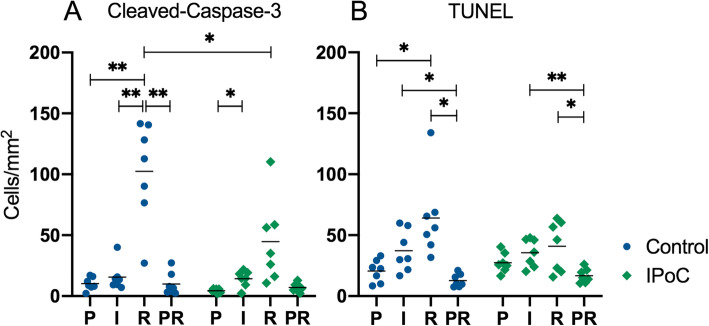
Cleaved-caspase- and TUNEL positive cell counts in the intestinal mucosa. Individual value plots of positive cell counts in cells/mm^2^ after immunohistochemical staining for cleaved-caspase-3 (**a**) and TUNEL (**b**) in the small intestinal mucosa from horses subjected to postconditioning (IPoC) and an untreated control group. The horizontal bar displays the mean. Significant differences are marked with an asterisk (* *p* < 0.05; ** *p* < 0.01). P = Pre-ischaemia, I = Ischaemia, R = Reperfusion, PR = proximal intestinal segment after reperfusion

To quantify the number of apoptotic cells in the mucosa, paraffin sections were immunohistochemically stained for cleaved-caspase-3. The cells exhibiting positive staining were enterocytes, stromal cells and inflammatory cells. During pre-ischaemia, the control group (group C) exhibited 10.2 ± 5.2 cleaved-caspase-3 positive cells/mm^2^, and group IPoC 4.4 ± 1.6 (Fig. 1a). During ischaemia, group IPoC showed a significant increase in positive cells (mean difference − 9.8 cells/mm^2^, CI − 19 to − 0.6, *p* = 0.04), without further progression during reperfusion. Group C showed a significant increase during reperfusion compared to pre-ischaemia (mean diff. -92.2 cells/mm^2^, CI − 140 to − 44, *p* = 0.002), ischaemia (mean diff. -86.8 cells/mm^2^, CI − 133 to − 41, p = 0.002), and the sample that was taken proximal to the ischaemic segment at the time point of reperfusion (Sample PR) (mean diff. 92.5 cells/mm^2^, CI 33 to 153, *p* = 0.006). There were no significant differences between both groups during pre-ischaemia and ischaemia; however, the cell count was significantly lower in group IPoC after reperfusion (mean diff. 57.7 cell/mm^2^, CI 1.2 to 114, *p* = 0.04). As additional marker for apoptosis and necrosis, a TUNEL assay was performed. During pre-ischaemia, the TUNEL positive cell count was 20.5 ± 9.3 cells/mm^2^ in group C, and 27.4 ± 8.1 in group IPoC (Fig. 1b). The increase in positive cells during ischaemia was not statistically significant. Group C exhibited a significant increase in the reperfusion sample compared to pre-ischaemia with a mean difference of 43.6 cells/mm^2^ (CI − 85.8 to − 1.4, *p* = 0.04). On the contrary, group IPoC did not show an increase during reperfusion. In both groups, the TUNEL positive cell counts were significantly lower in the PR samples compared to both ischaemia (group C: mean diff. 24.5 cells/mm^2^, CI 0.9 to 48, *p* = 0.04; group IPoC: mean diff. 18.8 cells/mm^2^, CI 6.9 to 31, *p* = 0.006) and reperfusion (group C: mean diff. 51.3 cells/mm^2^, CI 6.8 to 96, *p* = 0.03; group IPoC: mean diff. 24.1 cells/mm^2^, CI 3.3 to 45, *p* = 0.03). There were no significant differences between the groups for any of the time points.

### Inflammatory cells

**Fig. 2:**
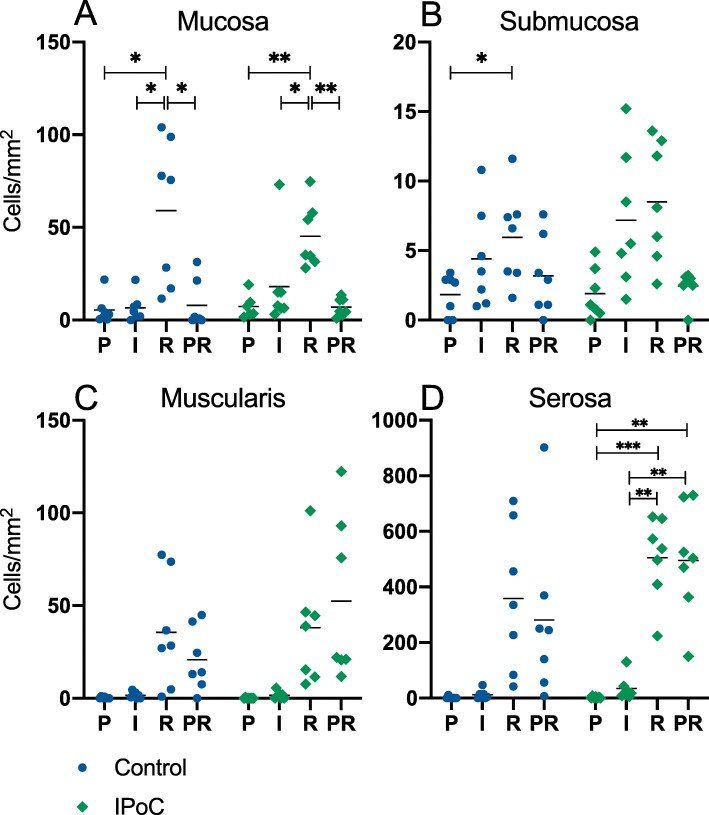
Calprotectin positive cell counts in the intestine. Individual value plots of the positive cell counts in cells/mm^2^ after immunohistochemical staining for cytosolic calprotectin in the mucosa (**a**), submucosa (**b**), muscularis (**c**) and serosa (**d**) of an untreated control group and a group subjected to IPoC. There were no significant differences between the groups. The horizontal bar displays the mean. Significant differences between the time points are marked with an asterisk (* *p* < 0.05; ** *p* < 0.01; *** *p* < 0.001). P = Pre-ischaemia, I = Ischaemia, R = Reperfusion, PR = proximal intestinal segment sampled after reperfusion

**Fig. 3:**
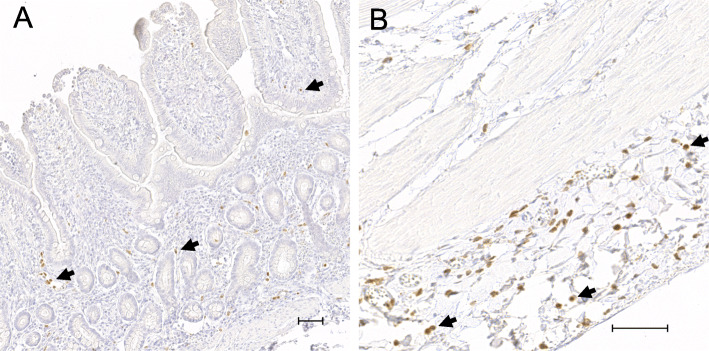
Calprotectin immunoreactivity in the small intestine. Microscopic images of immunohistochemical staining for cytosolic calprotectin in the mucosa (**a**) and serosa (**b**) from the same intestinal sample taken after reperfusion, illustrating the intense positive staining of inflammatory cells in these locations (arrows). The scale bar represents 50 μm

To assess the inflammatory cell count in the intestinal tissue, the sections were immunohistochemically stained for cytosolic calprotectin, which is present in neutrophils, monocytes and macrophages. There were no significant differences between the two groups for any of the time points or intestinal sections. In the mucosa of both groups, the reperfusion sample showed a significantly higher cell count compared to all other time points (Figs. 2 and 3). In the submucosa and muscularis propria, there were no significant changes over time, except for a higher submucosal cell count during reperfusion compared to pre-ischaemia in group C (mean diff. -4.1 cells/mm^2^, CI − 7.8 to − 0.5, *p* = 0.03). In the serosa, no significant differences could be detected between the time points in group C. However, group IPoC showed significantly higher cell counts in the reperfusion and PR sample, compared to both pre-ischaemia and ischaemia (Fig. 2).

### Heat shock Protein-70

**Fig. 4:**
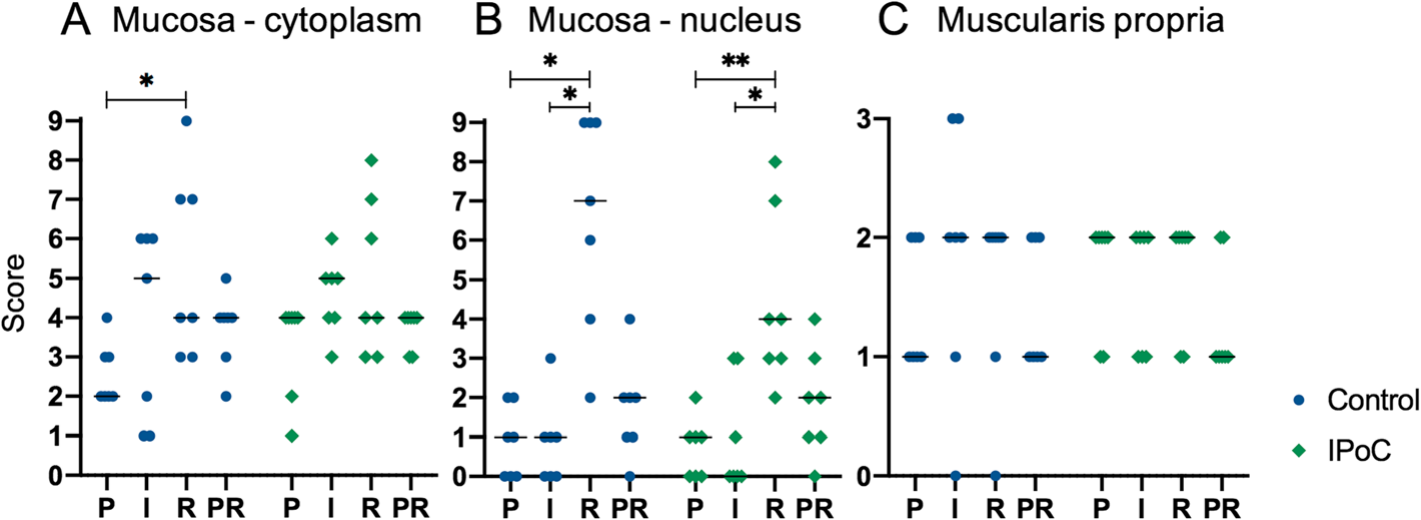
Heat Shock Protein-70 immunoreactivity score. Individual value plots of a semi-quantitative score assessing the immunoreactivity after immunohistochemical staining for Heat Shock Protein-70 of the small intestine from horses subjected to postconditioning (IPoC) and an untreated control group. The mucosal cytoplasm (**a**), the nuclei (**b**) and the muscularis propria (**c**) were scored separately. The horizontal bar displays the mean. Significant differences are marked with an asterisk (* *p* < 0.05; ** *p* < 0.01). P = Pre-ischaemia, I = Ischaemia, R = Reperfusion, PR = proximal intestinal segment after reperfusion

**Fig. 5:**
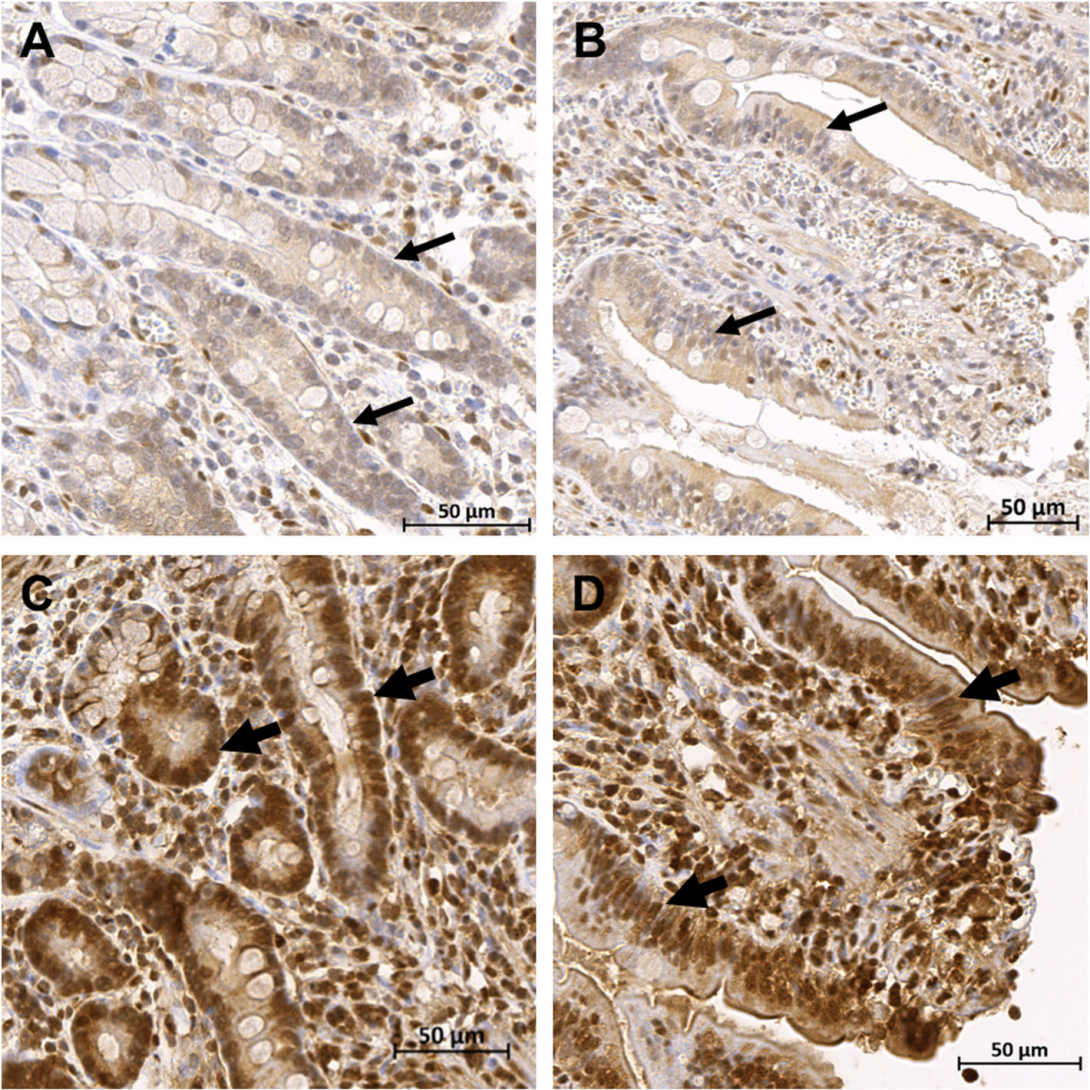
Heat Shock Protein-70 immunoreactivity in the small intestinal mucosa. Microscopic images of the crypts and villi of the small intestinal mucosa after immunohistochemical staining for Heat Shock Protein-70. Panels **a** and **b** are from an ischaemic sample, **c** and **d** are from the reperfusion sample of the same horse, illustrating an increase in immunoreactivity. Representative examples of the stained enterocyte nuclei are marked (narrow arrow for weak stained nuclei, broad arrows for the intensely stained nuclei), the enterocyte cytoplasm shows a mild to moderate staining

Immunohistochemistry for inducible HSP-70 was performed as an indicator for the heat shock response. Mainly enterocytes and inflammatory cells exhibited positive staining. During pre-ischaemia, the enterocytes of the crypts and the villus base exhibited no nuclear staining, and merely weak – mild staining of the cytoplasm, whereas the villus apex showed more staining of the enterocyte cytoplasm (1, 1–2) and nucleus (1, 0–2). There was no significant increase of the total cytoplasm and nucleus score during ischaemia (Fig. 4a). Yet reperfusion exhibited a significant rise in total nucleus score compared to both pre-ischaemia (*p* = 0.03 and 0.004 for group C and IPoC, respectively) and ischaemia (*p* = 0.02 for both groups) (Figs. 4b and 5). The total cytoplasm score did not significantly change during reperfusion. No differences between the treatment groups could be detected. When comparing the total cytoplasm score with the nucleus score for each time point, the cytoplasm staining was significantly higher than that of the nuclei at all time points except for the reperfusion time point. The staining of the muscularis was mild to moderate during pre-ischaemia, and did not change significantly over time (Fig. 4c).

### Myenteric plexus

**Fig. 6:**
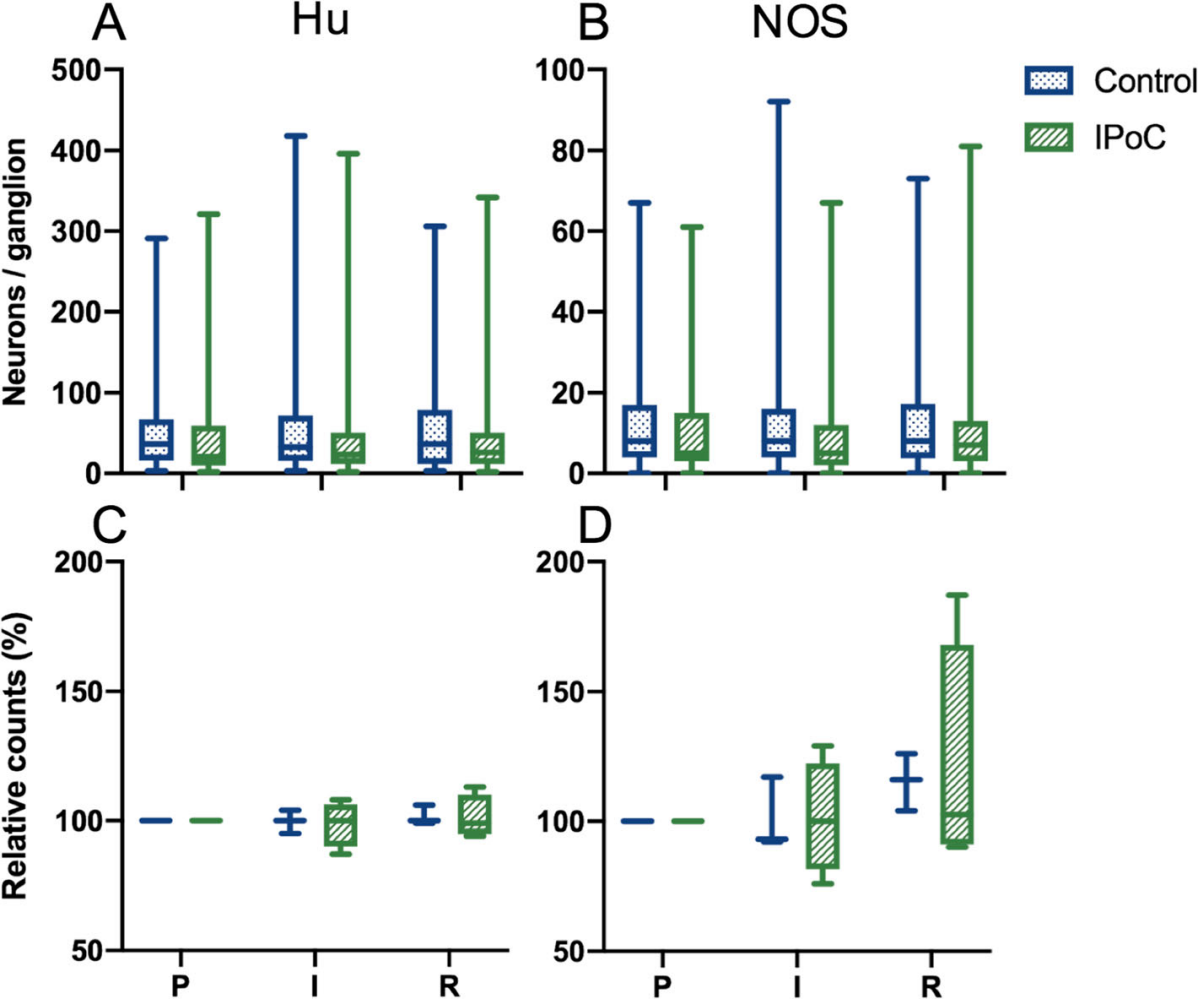
Hu and NOS stained neuronal counts in the myenteric plexus. Boxplot diagram of the Hu and NOS immunohistochemistry results. The left panels display the absolute and relative counts of the Hu stained neurons, and the right panels the NOS. The horizontal bar displays the median, the interquartile range is represented by the box, and the minimum and maximum by the whisker plots. IPoC = group undergoing postconditioning; P = pre-ischaemia; I = ischaemia; R = reperfusion

**Fig. 7:**
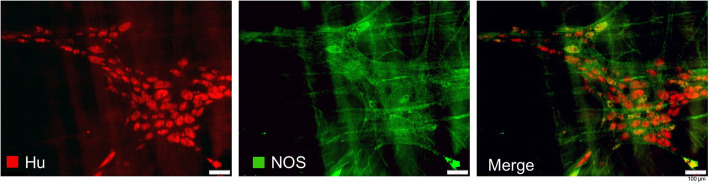
Hu and NOS immunoreactivity in the myenteric plexus. Fluorescence microscopy image of a ganglion in the myenteric plexus, displaying the cytoplasmic staining for Hu-positive neurons (red), and NOS-positive neurons (green). Scale bar 100 μm

To assess the neuronal cell count, Hu and NOS were immunohistochemically stained in a whole mount preparation of the myenteric plexus. Hu is an RNA binding protein commonly expressed in all enteric neurons, thus used as a pan-neuronal marker [31]. NOS is highly prevalent in interneurons and inhibitory motor neurons for the synthesis of nitric oxide as inhibitory neurotransmitter [9, 32]. A wide range in ganglion size was found, with a median of 37 (3–291) Hu-immunoreactive neurons in group C and 25 (2–321) in group IPoC (Fig. 6a and c). The NOS stained neurons exhibited a comparable variance, with 8 (0–67) positive neurons per ganglion in group C and 5 (0–64) in group IPoC (Fig. 6b and d), representing 21 and 22% of the total neuronal count, respectively. No changes could be detected after ischaemia or reperfusion, and there were no significant differences in absolute or relative cell counts between the groups (Fig. 6). The Hu- and NOS-positive staining was localized in the neuronal cytoplasm, and a translocation of Hu-immunoreactivity to the nucleus could not be observed in any of the samples (Fig. 7).

### Tissue levels of superoxide dismutase, myeloperoxidase and malondialdehyde

**Fig. 8:**
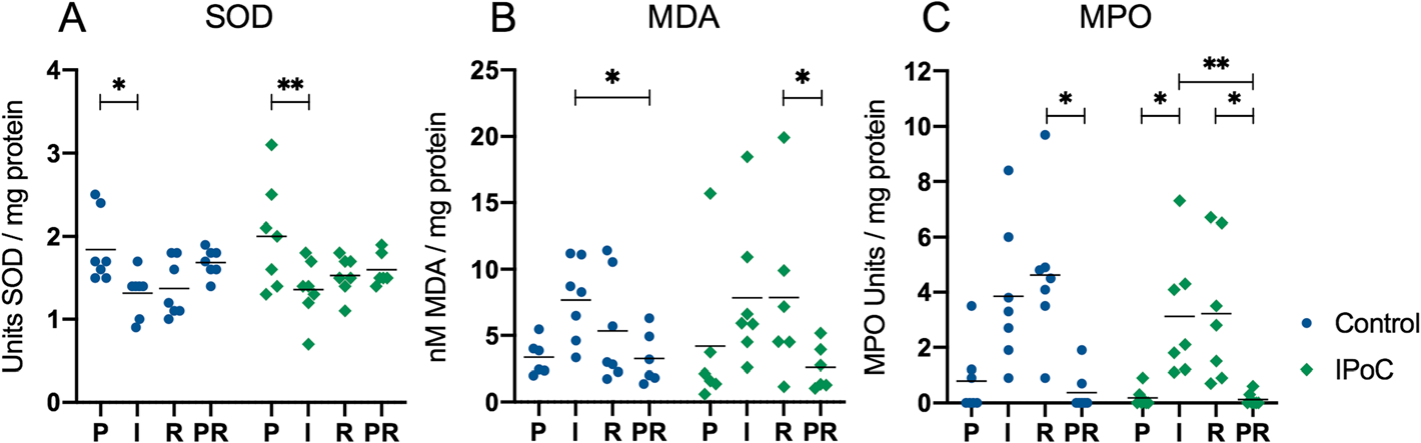
SOD and MPO activity and MDA content in the small intestine. Individual value plots of superoxide dismutase activity (**a**), malondialdehyde content (**b**), and myeloperoxidase activity (**c**) measured in full thickness intestinal tissue from horses subjected to postconditioning (IPoC) and an untreated control group. The horizontal bar displays the median. Significant differences are marked with an asterisk (* *p* < 0.05; ** *p* < 0.01;). P = Pre-ischaemia, I = Ischaemia, R = Reperfusion, PR = proximal intestinal segment after reperfusion

Superoxide dismutase (SOD) and malondialdehyde (MDA) as markers for oxidative stress and myeloperoxidase (MPO) as indicator for neutrophilic inflammation were determined in full thickness intestinal tissue samples including mucosa and serosa. For these variables, no differences could be detected between the groups for any of the time points. During pre-ischaemia, the SOD activity was 1.8 (± 0.4) and 2.0 (± 0.6) units/mg protein in group C and IPoC, respectively (Fig. 8a). A significant decrease was observed in both groups during ischaemia (group C: mean diff. 0.52, CI 0.11 to 1.0, *p* = 0.04; group IPoC: mean diff. 0.64, CI 0.13 to 1.2, *p* = 0.01), without further progression during reperfusion. MDA content was 3.4 (± 1.3) nM/mg protein in group C and 4.2 (± 5.7) in group IPoC during pre-ischaemia (Fig. 8b). No significant increase could be detected during ischaemia and reperfusion. The PR sample did show significantly lower values compared to ischaemia in group C (mean diff. 4.6, CI 0.2 to 8.9, *p* = 0.04) and compared to reperfusion in group IPoC (mean diff. 4.9, CI 0.3 to 9.5, *p* = 0.03). Barely any MPO activity was detected during pre-ischaemia, with a median of 0 (0–3.5) and 0 (0–0.9) units/mg protein for group C and IPoC, respectively (Fig. 8c). The activity was significantly higher during ischaemia in group IPoC, compared to pre-ischaemia (median diff. 2.1, *p* = 0.02) and the PR sample (median diff. 2.1, *p* = 0.004). In both groups, the PR sample showed significantly less MPO activity compared to the reperfusion segment (group C: median diff. 4.5, *p* = 0.04; group IPoC: median diff. 2.8, *p* = 0.04).

### Plasma levels of lactate, lactate-dehydrogenase and creatine-kinase

To determine the level of systemic oxidative stress, lactate, lactate dehydrogenase (LDH) and creatine kinase (CK) were measured in arterial (lactate) and venous (LDH and CK) blood samples. Pre-ischaemia, the lactate level was 1.1 (0.8–1.4), LDH 151 (123–206) and CK was 122 (88–180) mmol/l in group C. Plasma levels in group IPoC were 1.6 (0.9–2.2), 147 (122–241) and 125 (92–170) mmol/l, respectively. LDH and CK did not change significantly over time (Table 1). After reperfusion, lactate levels were significantly higher compared to pre-ischaemia in both groups (*p* = 0.01 and *p* = 0.005 for group C and IPoC, respectively). No differences between the treatment groups were detected.

**Table 1:** Blood oxidative stress variables during experimental ischaemia

			***Control***			***IPoC***		
			***P***	***I***	***R***	***P***	***I***	***R***
**Lactate**	Absolute	*Median*	1.1	1,9	1,9	1.6	2,15	3,05
		*Range*	0.8–1.4	1,1 - 2,3	1,3 - 2,6	0.9–2.2	1–3,1	2,1 - 4,2
	Relative	*Median*	100%	168%	217%*	100%	173%	235%**
		*Range*	–	119–360	121–414	–	112–299	191–312
**Lactate-dehydrogenase**	Absolute	*Median*	151	132	121	147	153	130
		*Range*	123–206	97–173	93–159	122–241	95–214	86–202
	Relative	*Median*	100%	100%	98%	100%	97%	101%
		*Range*	–	64–147	80–120	–	78–161	78–142
**Creatine-kinase**	Absolute	*Median*	122	108	103	125	104	111
		*Range*	88–180	64–169	83–165	92–170	76–126	84–122
	Relative	*Median*	100%	102%	99%	100%	98%	100%
		*Range*	–	65–124,4	93–138	–	70–162	85–178

Plasma levels of lactate, lactate-dehydrogenase and creatine-kinase were measured at the different time points (*P* Pre-ischaemia, *I* Ischaemia, *R* Reperfusion) in horses belonging to a control group (group C) and a group subjected postconditioning (group IPoC). The values are expressed as absolute value as mmol/l, and as percentage compared to the pre-ischaemia time point. There were no significant differences between the groups. Significant differences within the groups compared to the pre-ischaemia time point are marked with an asterisk (* *p* < 0.05; ** *p* < 0.01)

## Discussion

The main finding of this study is that the horses subjected to IPoC exhibited a lower mucosal apoptotic cell count indicated by fewer cleaved caspase 3 positive cells (Fig. 1). Moreover, in the control group the cell death progressed during reperfusion with a significant increase of both cleaved-caspase-3 and TUNEL positive cells, which could not be detected in the postconditioned group. This confirms our hypothesis that IPoC decreases apoptosis in the equine intestine after ischaemia/reperfusion injury. The inflammatory cell count (Fig. 2), mucosal HSP-70 staining (Fig. 4), blood lactate level (Table 1) and tissue SOD, MDA and MPO levels (Fig. 8) were all significantly affected by ischaemia and/or reperfusion. However, no differences between the treatment groups could be detected. Therefore, we rejected the hypotheses that IPoC would decrease inflammation, neuronal cell death and oxidative stress, and that IPoC would be accompanied by an increased heat shock response.

Intestinal IPoC studies in rat models have also demonstrated decreased levels of apoptotic markers in animals subjected to IPoC, reporting lower TUNEL positive cell counts [15–18, 33], and cleaved caspase-3 expression [15, 16]. A previous study in horses undergoing IPoC could demonstrate less epithelial denudation and lower paracellular permeability after experimental intestinal ischaemia [30]. These positive effects may be explained by the lower apoptosis cell count found in the current analysis, and may indicate a protective effect of IPoC on ischaemia reperfusion injury in horses. A potential mechanism of IPoC may involve the attenuation of apoptosis via the downregulation of PDCD4 and Fas-L [17], or the activation of JAK/STAT pathway [34]; however, these pathways were not investigated in the current study.

Intestinal ischaemia leads to hypoxia and concurrent oxidative stress [2]. In the investigated variables for oxidative stress, an effect of the experimental segmental ischaemia could be observed in the intestinal tissue analysis (Fig. 8). SOD activity was significantly decreased after ischaemia, indicating the presence of reactive oxygen species. Subjectively, the MDA level increased during ischaemia indicating increased lipid peroxidation; however, the only significant change over time was found between the PR sample and ischaemia (group C) or reperfusion (group IPoC). Looking at the blood analysis, only lactate levels were significantly higher during reperfusion (Table 1). Plasma LDH and CK were within reference values during all time points and did not change over time. Contrary to the significant changes in oxidative stress levels found in studies investigating IPoC in a rat model [14–16, 18–20, 33, 35], we found no differences between the treatment groups, contradicting an effect of IPoC on oxidative stress in the applied experimental model. A possible explanation may be that the low flow ischaemia implemented in this study elicited less oxidative stress than the complete occlusion of the cranial mesenteric artery (CMA) performed in the rat model. Regarding the systemic variables for oxidative stress, the intestinal segment subjected to segmental ischaemia may have been too short to elicit significant systemic effects. Moreover, the anaesthetic management of the horses with intravenous fluids and inotropic drugs also may have ameliorated the systemic effect. Smaller differences between the groups may not have been detected due to the relatively high variance between the samples combined with the low sample size. Individual variation between the horses may explain a higher variance compared to the relatively homogenous test population in rodent studies.

The serosa of the reperfusion and PR samples showed similar inflammatory infiltration by the presence of calprotectin positive cells (Fig. 2). In contrast, the mucosa did reveal a difference between these samples, exhibiting more inflammatory cells in the reperfusion sample compared to the PR sample. The inflammatory infiltrate in this proximal segment that has not been subjected to ischaemia, could represent an effect of the laparotomy and concurrent tissue manipulation [36, 37], or may be the result of remote intestinal reperfusion injury. The latter has also been found by other authors, reporting increased neutrophilic infiltration in the proximal resection margins of naturally occurring strangulating obstructions [38, 39]. This serosal influx of neutrophils is a normal site of entry into the intestinal wall after reperfusion injury [2, 38, 40, 41]. Only group IPoC showed a significantly higher calprotectin cell count in the serosa of the reperfusion and PR samples compared to the pre-ischaemia and ischaemia samples. The prolonged intestinal exteriorisation and manipulation necessary for performing IPoC, could possibly evoke a more pronounced neutrophilic response in the serosa. Another explanation may be that IPoC elicited a more severe inflammatory response due to reperfusion related injury. This could represent a potential disadvantage and a contraindication for performing IPoC in clinical cases. On the other hand, there were no significant differences in a direct comparison between the groups. Therefore, the relevance of these observations is questionable.

As additional marker for inflammation, MPO activity was quantified in the intestinal tissue (Fig. 8c), which has been shown to be lower in rats undergoing IPoC [15, 16, 18, 19]. This heme-enzyme is predominantly present in neutrophils, but is also expressed in monocytes and macrophages [42, 43]. The MPO activity in the intestinal tissue was significantly lower in the proximal intestinal segment compared to the reperfusion segments. Comparing this to the calprotectin positive cell counts, this may reflect the lower cell count in the mucosa of the proximal samples. As with the calprotectin positive cell counts, no difference was found between the groups. This suggests that IPoC does not significantly alter the inflammatory infiltrate in the current model, and that the reduced apoptosis level after IPoC is not mediated through a decreased neutrophilic response.

The neuronal count in the myenteric plexus was not significantly affected by ischaemia or reperfusion (Fig. 6), possibly related to a relatively short timeframe of the current study. Studies investigating the effect of experimental small intestinal ischaemia in rodents, have shown that neuronal loss was not apparent before 24 h of reperfusion [44], and that apoptosis of enteric neurons detected by TUNEL stain could not be found before 6 h [32, 45]. Evaluating the myenteric plexus in the cleaved-caspase-3 and TUNEL stained intestinal sections, no immunoreactivity of the myenteric plexus was found. A more short term marker of cell damage is the change in the distribution of Hu immunoreactivity towards the nucleus, which has been detected as early as 1 h post-ischaemia [32]. We could not observe any nuclear Hu staining, possibly indicating the duration of reperfusion might not be the only cause for the absence of detectable ischaemia reperfusion injury to the enteric neurons. The application of different ischaemia models could also account for this disparity. Nitrergic neurons have been reported to account for 23–52% of all myenteric neurons in rodents [9, 31], with an increased NOS ratio after 48 h of reperfusion in response to ischaemia [32]. The basal NOS ratio in the current study was slightly lower at 21–22%, without any changes after ischaemia or reperfusion. The submucosal neuronal plexus may have responded differently; however, this was not investigated in the current study.

The highly stress-inducible HSP-70 has been shown to guide co-translational folding for protein maturation and to restore folding after injury, thereby promoting cell survival [46]. This is the first report of the heat shock response in experimental equine small intestinal ischaemia. Pre-ischaemia, HSP-70 was predominantly located in the cytoplasm, and exhibited an increase in nuclear immunoreactivity during reperfusion (Fig. 4). This nuclear shift has been reported in clinical cases of acute intestinal ischaemia in humans and horses [47, 48]. Cell culture experiments have found that HSP-70 translocates to the nucleus during cellular stress, and relocates to the cytoplasm as the cell recovers [49, 50]. This is considered as part of the stress response and possibly promotes cell survival by maintaining or restoring the epigenetic regulating proteins in the nucleus [46, 50, 51]. In the equine study, increased HSP-70 immunoreactivity of the intestinal segment proximal to the strangulating obstruction was not related to the clinical outcome [47]. Several studies investigating ischaemic preconditioning in laboratory animals, have implicated that increased inducible HSP-70 expression contributes to the protective mechanism of preconditioning [27–29, 52]. However, we could not detect an effect of IPoC on the expression or distribution of this protein.

Limitations of the current study are the small sample size and high variance in some of the data sets, possibly precluding the detection of smaller differences. Furthermore, there was no direct comparison with reperfusion injury. As mentioned previously, the duration of ischaemia and reperfusion may have been too short for complete evaluation of some of the tested variables. Moreover, the long-term effect of IPoC could not be assessed. The duration of the experimental ischaemia and reperfusion was limited, because extended anaesthesia times in horses can compromise cardiovascular stability and induce muscular damage and inflammation. Letting the horses recover from anaesthesia prior to euthanasia was considered unethical. Pharmacological preconditioning with xylazine and isoflurane administered for anaesthetic premedication and maintenance, may have mitigated the potential effect of the ischaemic injury or IPoC. However, this effect was present in both groups. A limitation of the TUNEL assay is that it may identify both apoptotic and necrotic cells by labelling double stranded DNA breaks that are produced during early necrosis [53–55], complicating the interpretation of the results. Cleaved caspase-3 is specific for apoptosis, yet this effector caspase does not indicate through which pathway the apoptosis was initiated [56]. Therefore, the quantification of early initiator caspases or markers for cell necrosis could have provided more clarity on the mechanism of cell death. Another limitation, is that the difference in ischaemia model precludes the direct comparison with previous reports investigating IPoC in a rat model. Nevertheless, the segmental jejunal ischaemia model is more representative for clinical strangulating colic in horses. The CMA model is used as a model for human intestinal ischaemia, which is suitable for the more generalized obstruction of the cranial mesenteric artery described in humans [57]. For clinical cases with local strangulating obstructions such as small bowel volvulus and incarcerating hernias, the current animal model may be more appropriate [58].

## Conclusion

Fewer apoptotic cells were found in the mucosa of the horses subjected to IPoC. This could indicate a protective effect on small intestinal ischaemia reperfusion injury in horses, yet this cannot be concluded without direct comparison with histomorphological injury. None of the other tested variables were significantly affected by IPoC, suggesting only a limited impact. These negative results are in contradiction with the findings of many rodent studies investigating IPoC in small intestinal ischaemia. This disparity may be caused by a difference in species, anaesthetic protocol, the applied ischaemia model and the degree of ischaemic injury. Therefore, further research on the mechanism of action of IPoC in equine segmental ischaemia and its effect in clinical cases of strangulating colic is needed. Although not significantly affected by IPoC, the observed increase in nuclear HSP-70 after reperfusion suggests that this molecular chaperone may be of relevance in the equine intestinal response to ischaemia, warranting further investigation.

## Methods

### Experimental design

The study was reviewed by the Ethics Committee for Animal Experiments of Lower Saxony, Germany, and approved according to §8 of the German Animal Welfare Act (LAVES 33.8–42,502–04-18/2856). A power analysis was performed prior to commencing the study using free available software (G*Power 3.1.9.2, *Heinrich Heine Universität, Düsseldorf, Germany*). To detect a difference of 25 cells/mm^2^ in the immunohistochemistry cell counts with a standard deviation of 15 cells/mm^2^, a sample size of 7 horses per treatment group was required, based on a power of 0.8 and alpha of 0.05. Fourteen horses, owned by the university, were assigned to a group subjected to ischaemic postconditioning (group IPoC; *n* = 7) and a control group (group C, n = 7) using simple randomisation with an equal allocation ratio.

### Animals

Group C consisted of five Warmbloods, one Islandic horse and one Thoroughbred, with a mean age of 12.6 ± 8.7 years and mean weight of 535 ± 89 kg. Group IPoC consisted of four Warmbloods, one Islandic pony, one Thoroughbred, and one Standardbred, with a mean age of 10.4 ± 8.6 years and weight of 506 ± 96 kg. All horses were systemically healthy, and had been elected for euthanasia due to severe orthopaedic problems. Faecal egg counts were done, and all were below the cut-off value of 200 eggs per gram. The horses were stabled at the facilities of the equine clinic at least 2 weeks prior to surgery. No medication was administered during this time. The horses had free access to hay and water and were hand walked daily. Prior to anaesthesia, feed but not water was withheld for 6 h.

### Anaesthesia and surgical procedure

After premedication with 0.7 mg/kg body weight (BW) xylazine (Xylavet 20 mg/ml, *CP-Pharma GmbH, Burgdorf, Germany*), general anaesthesia was induced with 0.1 mg/kg BW diazepam (Ziapam 5 mg/kg, *Ecuphar GmbH, Greifswald, Germany*) and 2.2 mg/kg ketamine (Narketan, *Vétoquinol GmbH, Ismaning, Germany*). Anaesthesia was maintained with isoflurane (Isofluran CP, *CP-Pharma GmbH*) in 100% oxygen, and continuous rate infusions with lactated Ringer’s solution (Ringer-Laktat EcobagClick, *B. Braun Melsungen AG, Melsungen, Germany*) and dobutamine (Dobutamin-ratiopharm 250 mg, *Ratiopharm GmbH, Ulm, Germany*) were given to effect, to maintain the mean arterial blood pressure between 60 and 80 mmHg. A routine pre-umbilical median laparotomy was performed in dorsal recumbency. Segmental small intestinal ischaemia was induced in 1.5 m jejunum by occlusion of the mesenteric arteries and veins with umbilical tape. The ligature was tightened under monitoring of the intestinal microperfusion with microlightguide spectophotometry and laser Doppler fluxmetry (O_2_C, *LEA Medizintechnik GmbH, Giessen, Germany*), and the ligature was tied as soon as the blood flow was reduced by 90%. The ischaemia was maintained for 90 min. In between the different stages of the experiment, the intestines were replaced in the abdominal cavity and the laparotomy was temporarily closed with towel clamps. In group C, the ligature was released without manipulation of the vessels or the intestine. In group IPoC, postconditioning was implemented after release of ischaemia by clamping the mesenteric vessels with large haemostatic forceps, with the jaws covered with latex catheters to decrease trauma to the vessel walls. This elicited complete vascular occlusion as confirmed by laser Doppler Fluxmetry. Clamping was performed for 3 cycles of 30 s, alternated with 30 s of reperfusion, which is the most commonly reported intestinal IPoC algorithm [16–18, 21, 59]. Following ischaemia, a reperfusion duration of 120 min was implemented in both groups. Subsequently, the horses were euthanized by intravenous administration of 90 mg/kg pentobarbital (Release 50 mg/ml, *WDT eG, Garbsen, Germany*) without regaining consciousness. Everyone involved in the experimental trial was aware of the group allocation of the horses during the conduct of the experiment.

### Sample collection

Full thickness intestinal segments of 10 cm jejunum were taken just before induction of ischaemia (pre-ischaemia sample, P), at the end of ischaemia (ischaemia sample, I), and at the end of reperfusion (reperfusion sample, R). At this time point, an additional intestinal sample was taken from the segment just proximal (orad) to the post-ischaemic segment (proximal sample, PR). The lumen of the remaining intestinal segments was occluded with umbilical tape and lavaged prior to replacement in the abdominal cavity. Two antimesenterial sections of 2 cm^2^ from each sample were fixed in a 4% formaldehyde solution and subsequently embedded in paraffin following standard procedure. Smaller sections of full thickness tissue from the antimesenterial intestine were snap frozen in liquid nitrogen, and stored at − 80 °C until further processing. Blood samples were taken from the jugular vein and the facial artery at the end of the pre-ischaemic, ischaemic, and reperfusion periods and collected in heparinised tubes.

### Immunohistochemistry

To quantify the number of apoptotic and necrotic cells, paraffin sections were immunohistochemically stained for cleaved-caspase-3 (dilution 1:200, rabbit-anti-human, CleavedCaspase-3Asp175 antibody, *Cell Signalling Technology Europe B.V., Leiden, The Netherlands*), and terminal deoxynucleotidyl transferase dUTP nick end labelling (TUNEL) (ApopTag® Peroxidase In Situ Apoptosis Detection Kit, *Merck KGaA, Darmstadt, Germany*) was performed as described previously [60]. Furthermore, sections were stained for cytosolic calprotectin using monoclonal mouse anti-human myeloid/histiocyte antigen (clone MAC 387, *DakoCytomation, Glostrup, Denmark*) as described elsewhere [61]. Immunohistochemical staining for HSP-70 was also performed. In short, the slides were demasked by heating in citrate solution, followed by blocking with 20% goat serum. The slides were incubated overnight with 1:400 polyclonal rabbit antibody against inducible HSP-70 (Anti-Hsp70 antibody ab79852, *Abcam, Cambridge, UK*), followed by incubation with secondary antibody (1:200 goat-anti-rabbit) and then the ABC reagent (Vectastain ABC, *Biozol diagnostics Vertrieb GmbH, Eching, Germany*). The negative control was incubated with 1:2000 rabbit serum (R4505, *Sigma Aldrich Merck KGaA, Darmstadt, Germany*) instead of the primary antibody. Equine testicular tissue was used as a positive control. Further processing was performed with the same protocol as described for the other stainings.

All slides were scanned to a digital format at 20x magnification (Axio Scan.Z1, *Carl Zeiss GmbH, Oberkochen, Germany*), and subsequently evaluated using the accompanying software (Zen Blue 3.0, *Carl Zeiss GmbH*). One section per sample was evaluated by one observer (NV trained by MHT and CP), who was blinded to the identity of the slides. For the cleaved-caspase-3 and TUNEL stained slides, the positive cell count in the mucosa was determined. The calprotectin positive cells were counted separately in the mucosa, submucosa, muscularis and serosa. The surface area of a section of 10 to 15 villi was determined in mm^2^. Following manual counting of the positive cells, the cell counts were expressed in cells/mm^2^.

Due to the more diffuse staining pattern in the HSP-70 stained slides, a semi-quantitative score for enterocyte immunoreactivity was developed. The enterocyte cytoplasm and nuclei were graded separately for staining intensity (0 – absent to very weak, 1 – low, 2 - moderate, 3 – intense) in the crypts, the villus base and villus apex, at a standard fixed colour setting for all sections. For further analysis, the cytoplasm scores (0–3) were added up, resulting in a total mucosa cytoplasm score (0–9) per section, and the same was done for the nucleus scores. The muscularis propria was given one grade for its cytoplasmic staining intensity. The nuclei in the muscularis were not scored separately, since these exhibited a similar staining intensity throughout all the sections.

In 7 horses (3 in group C, 4 in group IPoC), additional immunohistochemical staining for Hu and nitric oxide synthase (NOS) was performed to evaluate the number, proportion and morphology of the neurons in the jejunal myenteric plexus in samples P, I and R. The full thickness intestinal tissue was fixed in a solution containing 4% paraformaldehyde and 0.002% picric acid in 0.1 mol/l phosphate buffer overnight at 4 °C. Subsequently, a whole mount preparation of the myenteric plexus was performed removing the different intestinal layers by careful manual dissection. Then the tissue was incubated for 1 h in phosphate buffered saline (PBS)/NaN3 (0.1%)/horse serum (HS, 4%) (*Sigma Aldrich, Darmstadt, Germany*) to avoid unspecific staining. After this, the tissue was incubated 48 h with the primary antibodies (Mouse α Hu-Biotin 1:50*, Alexis, San Diego, USA*; Rabbit α NOS 1:3000, *Molecular Probes, Eugene, USA*). Subsequently, the tissue was washed (3 × 10 min) in phosphate buffer and then incubated for 12 h with the secondary antibodies (Donkey α rabbit Cy2 1:200, Streptavidin Cy3 1:500, *Jackson ImmunoResearch, Cambridgeshire, United Kingdom*). Finally, specimens were washed in PBS, mounted on poly-l-lysine-coated slides and cover slipped with a solution of PBS (pH 7.0) /NaN_3_ (0.1) containing 65% glycerol. The preparations were examined with an epifluorescence microscope (*Olympus IX70, Olympus, Hamburg, Germany*), equipped with appropriate filter blocks. Images were acquired and analysed with a monochrome camera (XM 10; *Olympus*) using the Olympus cellSens standard software. The neuronal count was performed in a blinded manner. Neurons were counted in 50 ganglia per sample. The neuronal counts of the individual horses were grouped based on the time point and test group. To adjust for the high variability between the horses, relative cell counts of the ischaemia and reperfusion sample were determined per horse as the percentage of the pre-ischaemia sample mean. Furthermore, the percentage of NOS immunoreactive neurons compared to the total neuron (Hu) count was determined.

### Enzymatic assays

The full thickness intestinal tissue was homogenised in assay specific lysis buffers as described below using a high-speed homogenisator (FastPrep-24™ 5G, *MP Biomedicals Germany GmbH, Eschwege, Germany*). Protein content of each individual homogenized sample was assessed by performing a Bradford assay, as described previously [62].

The SOD activity was detected using a commercially available colorimetric assay kit (19,160 SOD Determination Kit, *Sigma Aldrich/Merck KGaA*) after homogenisation in an appropriate lysis buffer (CB cell lysis buffer, *Cell Biologics, Chicago, USA*). The SOD activity assay relies on the formation of formazin dye (absorbance at 450 nm), when tetrazolium salt is reduced with a superoxide anion. Bovine SOD (Superoxide Dismutase Bovine Erythrocytes, *Merck KGaA*) was used to establish a concentration curve. MPO activity was determined based on its ability to catalyse the formation of hypochlorous acid using a commercially available assay kit, which included lysis buffer and positive controls (Myeloperoxidase Colorimetric Activity Assay Kit, *Sigma Aldrich/Merck KGaA*). The MDA content was determined by measuring the colorimetric (532 nm) product that forms when MDA reacts with thiobarbituric acid, with an assay kit including lysis buffer and positive controls (Lipid Peroxidation Assay Kit, *Sigma Aldrich/Merck KGaA*). The kits were used in accordance with manufacturer’s instructions, with only a small modification in the MDA assay. Here, butanol (1:2 of total volume) was added for the purification of the samples, and the colorimetric measurements were performed on the organic layer, without prior evaporation of the butanol.

All assays were analysed using a microplate spectrophotometer (Epoch, *Biotek Germany, Bad Friedrichshall, Germany*). To correct for the variation in protein content between the individual samples after homogenisation, all values were expressed as units (SOD or MPO activity) or nMol (MDA levels) per mg protein in the sample. The assays were performed in duplicates and the mean of both values was used for further analysis. Concentration curves were plotted by use of commercially available software (Graphpad Prism 8.4.2, *Graphpad Software Inc., San Diego, California, USA*), and a r^2^ value of > 0.97 was considered appropriate. In the MPO measurements the negative values close to zero, were set to zero to enable statistical analysis.

### Blood analysis

LDH and CK were measured in plasma immediately after sampling by use of a commercially available analyser (cobas c311, *Roche/Hitachi, Mannheim, Germany*). Arterial blood samples for lactate measurement were taken at the same time and analysed immediately (ABL825 flex, *Radiometer Medical ApS, Bronshoj, Denmark*). For comparison between the time points and groups, a correction for haemodilution was made by measuring albumin in the corresponding samples and expressing the values as mmol/g albumin. Subsequently, the ischaemia and reperfusion samples were expressed as relative values compared to pre-ischaemia.

### Data analysis

Statistical analysis and graph design were performed using commercial software (Graphpad Prism 8.4.2, *Graphpad Software Inc., San Diego, California, USA*). Normal distribution was assessed with the Shapiro Wilks test and by visual inspection of QQ plots of the model residuals. The normal distributed data were expressed as mean (± SD), and data that did not show normal or lognormal distribution, were expressed as median (min-max). The equality of variances was tested by visual assessment of the homoscedasticity plots, and by performing Levene’s test. Statistical significance was set at *p* < 0.05.

For analysis of the normally distributed data, a two-way repeated measures ANOVA was performed for one independent effect (group), and the time points as repeated effect. This was implemented to compare the values between the different time points and groups, with the horses as subject effect. The Geisser-Greenhouse correction was applied for the *p*-values. Multiple pairwise comparisons were performed with a post-hoc Tukey test to compare the different time points within the groups, and a post-hoc Sidak test for group comparison.

For the normally distributed SOD and MDA results, the ROUT outlier test was implemented with the maximum desired False Discovery Rate (Q) set at 1%. After testing for equality of variance, mixed effect model fitted as a two-way repeated measures ANOVA for missing values was performed for one independent effect (group), and the time points as repeated effect. Post hoc testing was performed as described above.

For the ordinal and not normally distributed data (HSP-score, MPO units), distribution free nonparametric models were used for independent (treatment and control group) and correlated (time points) effects. A Mann-Whitney-U test was executed to compare the results between the different groups at each time point. For comparing the correlated different time points, a Friedman test in combination with the post hoc Dunns-test for multiple pairwise comparisons were performed.

## Data Availability

The datasets analysed during the current study are available in the Mendeley repository, openly available under the following reference: Verhaar, Nicole (2020), “Ischaemic Postconditioning in Equine Jejunal Ischaemia”, Mendeley Data, 10.17632/mxhhxpvpvj.2
